# Environmental Exposure to Triclosan and Semen Quality

**DOI:** 10.3390/ijerph13020224

**Published:** 2016-02-17

**Authors:** Wenting Zhu, Hao Zhang, Chuanliang Tong, Chong Xie, Guohua Fan, Shasha Zhao, Xiaogang Yu, Ying Tian, Jun Zhang

**Affiliations:** 1 School of Public Health, Shanghai Jiao Tong University School of Medicine, Shanghai 200025, China; windy5490@163.com (W.Z.); tianmiejp@163.com (Y.T.); 2 MOE and Shanghai Key Laboratory of Children’s Environment Health, Xinhua Hospital, Shanghai Jiao Tong University School of Medicine, Shanghai 200092, China; haozhang18@foxmail.com (H.Z.); zhaosha2012@126.com (S.Z.); xiaogangrain@sina.com (X.Y.); 3 Shanghai Key Laboratory of Kidney and Blood Purification, Shanghai 200032, China; 4 Department of Science and Research, International Peace Maternity and Child Health Hospital of China Welfare Institute, Shanghai 200030, China; tcl_sh@aliyun.com; 5 Department of Andrology, International Peace Maternity and Child Health Hospital of China Welfare Institute, Shanghai 200030, China; philip_1981@hotmail.com (C.X.); fanyishi166@sina.com (G.F.)

**Keywords:** cross-sectional study, semen quality, triclosan, endocrine disruptor

## Abstract

Triclosan (2,4,4′-trichloro-2′-hydroxy-diphenyl ether, TCS) is widely used in personal care, household, veterinary and industrial products. It was considered as a potential male reproductive toxicant in previous *in vitro* and *in vivo* studies. However, evidence from human studies is scarce. Our study aims to investigate the relationship between TCS exposure and semen quality. We measured urinary TCS concentrations in 471 men recruited from a male reproductive health clinic. TCS was detected in 96.7% of urine samples, with a median concentration of 0.97 ng (mg·creatinine)^−1^ (interquartile range, 0.41–2.95 ng (mg·creatinine)^−1^). A multiple linear regression analysis showed a negative association between natural logarithm (Ln) transformed TCS concentration (Ln-TCS) and Ln transformed number of forward moving sperms (adjusted coefficient β = −0.17; 95% confidence interval (CI) (−0.32, −0.02). Furthermore, among those with the lowest tertile of TCS level, Ln-TCS was negatively associated with the number of forward moving sperms (β = −0.35; 95% CI (−0.68, −0.03)), percentage of sperms with normal morphology (β = −1.64; 95% CI (−3.05, −0.23)), as well as number of normal morphological sperms, sperm concentration and count. Our findings suggest that the adverse effect of TCS on semen quality is modest at the environment-relevant dose in humans. Further studies are needed to confirm our findings.

## 1. Introduction

Triclosan (2,4,4′-trichloro-2′-hydroxy-diphenyl ether, TCS), a broad spectrum antimicrobial agent, is widely used in personal care, household as well as health care products, including toothpastes, antibacterial soaps, shampoos, deodorants, cosmetics, kitchen utensils, toys, bedding and clothes [1]. The use of these products results in a large amount of triclosan entering sewage water and thus the ecosystem. TCS has been detected in surface water, sediment, soil, biosolids and aquatic species [2,3].

The daily contact of all types of TCS-containing products and the environment contributes to the wide spread exposure of TCS to human beings’. The derma and digestive tract are two main positions of TCS absorption [4,5]. TCS has been detected in diverse human bodyfluids and tissues [6,7,8], and 74.6% of samples had TCS detected at concentrations of 2.4–3790 mg·μL^−1^ in 2003–2004 National Health and Nutrition Examination Surveys (NHANES) of the U.S. population [8].

Despite its high occurrence across the ecosystem, the health effect of TCS has not been well studied. Recently, TCS is suspected to be a potential male reproductive toxicant. *In vitro* studies have shown that TCS binds to androgen receptors (AR) and exhibits an anti-androgenic activity in human breast cancer cells [9]. This chemical reduces the production of testosterone in Leydig cells and disturbs the function of major steroidogenic enzymes [10,11]. TCS has been shown to decrease weights of the testes and sex accessory organs, followed by decrease in sperm density [12]. TCS has also exhibited a tendency to accumulate in the epididymis [13]. However, human studies on the effect of TCS on male reproductive health are few to date. In this study, a cross-sectional investigation was carried out to evaluate the association between TCS exposure measured by urinary TCS concentration and semen quality in humans.

## 2. Materials and Methods

### 2.1. Study Subjects

Male volunteers were recruited at a male reproductive health clinic in a university affiliated teaching hospital during November 2013–March 2014. The study was approved by the Ethics committee of Xinhua Hospital Affiliated to Shanghai Jiao Tong University School of Medicine, Shanghai, China (XHEC-C-2013-001). All participants signed a written informed consent. Through a face-to-face interview, a trained interviewer filled in a standardized questionnaire to obtain information on participants’ demographic characteristics, living and working environment, health-related behaviors, sexual and reproductive status, as well as medical history that may affect reproductive health. The participants were asked to provide a single spot urine sample and semen specimen as part of their clinical examination on the day of their clinic visit.

**Figure 1:**
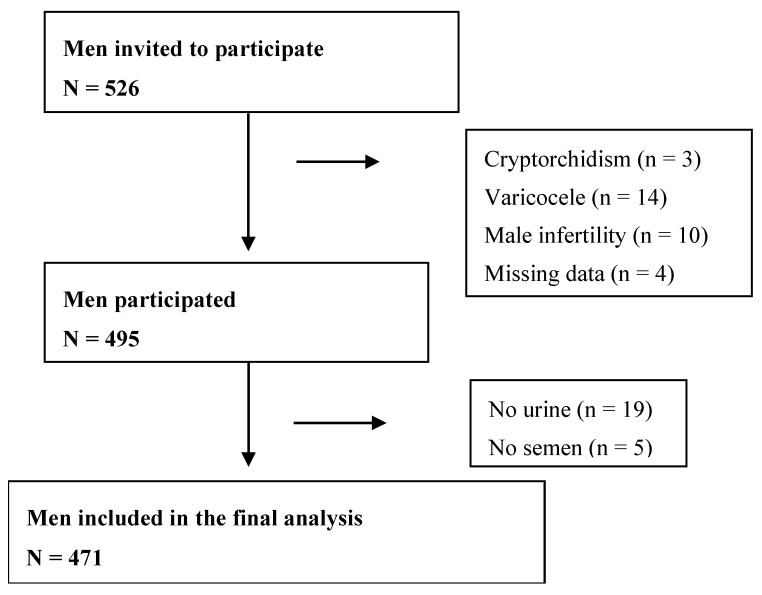
The inclusion and exclusion of participants in the study.

Of the 526 men recruited, we excluded subjects with cryptorchidism, varicocele, male infertility and those who failed to provide urine or semen samples. Four-hundred-seventy-one men were included in the final analysis (Figure 1).

### 2.2. Measurement of Urinary TCS

About 50 mL urine sample was collected in a sterile polypropylene cup from each subject, aliquoted to polypropylene storage tubes (15 mL, 430791 Corning CentriStar) and stored at –80 °C until TCS analysis.

Total urinary TCS (free and conjugated) concentration was measured in each urine sample using internal standard method as described previously [14]. After the samples were thawed at 4 °C, 10 μL internal standard TCS-D3 (2 μg·mL^−1^, Dr. Ehrenstorfer GmbH, Augsburg, Germany) was added into a 4 mL urine sample. Then, samples were incubated in 1 mol·L^−1^ ammonium acetate buffer solution (pH = 5.0) with 20,000 IU·mL^−1^ β-glucuronidase (TypeH-1 from Helix pomatia, Sigma-Aldrich, St. Louis, MO, USA) at 37 °C overnight to hydrolyze the conjugated TCS. Secondly, regeneration of solid phase extraction (SPE) (500 mg (3 mL)^−1^, Supelco, Bellefonte, PA, USA) with 3 mL 100% methanol and equilibration of SPE with 3 mL H_2_O were performed. Thirdly, TCS was retained and concentrated by a SPE, eluted by 9 mL acetonitrile, concentratedby speed vacuum concentrator and redissolved in 200 uL 90% methanol. After that, separation and determination of TCS were performed using high performance liquid chromatography–electrospray ionization tandem mass spectrometry (HPLC-MS/MS) (Agilent 1290-6490, Agilent Technologies, Little Falls, DE, USA). Separations were carried out on a ZORBAX RRHD Eclipse plus C18 (100 × 2.1 mm, 1.8 μm) column (Supelco, Bellefonte, PA, USA). The column was maintained at 40 °C. The mobile phase consisted of (A) 10 mmol/L ammonium acetate in water and (B) methanol. The gradient elution method was as follows: 60% B at 0–5 min (0.3 mL·min^−1^), 90% B at 6–9 min (0.3 mL·min^−1^), then changed gradually to 50% B and ended at 10 min (0.3 mL·min^−1^). The samples were injected with a 5 μL loop. Samples were analyzed in multiple reaction monitoring (MRM) mode. The optimized MS/MS parameters were as follows: electrospray ionization (ESI), ion spray voltage, 3000 V and temperature, 200 °C.

The quality control (QC) materials were prepared with TCS standard (Dr. Ehrenstorfer GmbH, Augsburg, Germany) and a blank urine which was pooled by TCS undetectable samples obtained from several anonymous donors. The limit of detection (LOD) was 0.1 μg·L^−1^. Linearity was valid over the range of 0.1–50 ng·mL^−1^ (*r*^2^ = 0.998). Low, moderate and high concentration quality control materials were 0.8 ng·mL^−1^, 8 ng·mL^−1^ and 40 ng·mL^−1^, which were analyzed five times in a single day, replicating these determinations on five consecutive days. All the intra- and inter-batch precisions were less than 15%. The recovery was 91.1%. The same three quality controls of low, moderate and high concentration were also analyzed in parallel with the other 23 unknown samples in each analytical batch to assure the accuracy of determination. Analysts were blinded to all information during the test. We also measured creatinine (CR) concentration in each urine sample using enzymatic method on automatic chemical analyzer (7100 Automatic Analyzer, Hitachi, Japan) to correct the fluctuations of TCS levels caused by urine concentration or dilution.

### 2.3. Semen Analysis

Semen was collected in a private room by masturbation into a sterile polypropylene cup, and was analyzed within 60 min after collection. After liquefaction of the semen at 37 °C, semen volume was measured with a serologic pipette. Semen quality parameters including concentration, motility, and speed were analyzed by using a computer-aided semen analyzer (SQA-V, Medical Electronic System, Hatavorzo, Israel) in accordance with the World Health Organization (WHO) guidelines [15]. Total sperm count (10^6^ per ejaculate) was calculated by semen sample volume (milliliters) multiplied by the sperm concentration (10^6^ mL^−1^). For speed, only the average path velocity was measured. For morphological evaluation, 10 µL semen was spread onto a glass slide and air-dried at room temperature. The smears were then stained with Giemsa stain and sperm morphology was assessed by the same two professional technicians, according to WHO criteria [15].

### 2.4. Statistical Analysis

All data were doubly entered into the EpiData database. Urine TCS values less than LOD were imputed as LOD × 2^−0.5^, a commonly accepted practice [16,17]. The data analysis was performed using SAS version 9.2 (SAS Institute Inc., Cary, NC, USA). The level of statistical significance was set at 0.05.

Factors that may affect the relationship between TCS exposure and semen quality biologically were chosen as potential confounders [18]. Age, smoking status, education and household income have been identified as predictors of TCS exposure [19]. For analyses of semen parameters [20,21], body mass index (BMI), drinking status, abstinence period were also included in the multiple linear model. Additionally, exposure history of other environmental chemicals or heavy metals (Table S1) were adjusted to examine the independent effect of TCS on semen quality. Age and BMI were modeled as continuous variables, whereas other variables were included in the model as categorical variables. To improve interpretability, all models were adjusted for the same covariates.

The continuous variables were compared with the independent sample *t*-test for those that were normally distributed or the Wilcoxon rank-sum test for non-normally distributed variables. The chi-square test was used to examine differences in categorical variables. General linear regression was used to examine the correlation between the urine TCS level and various semen quality parameters adjusted for potential confounders. The variables of skewed distribution were included in the model after natural logarithm transformation. Box plot and smoothing spline were applied to depict the relation of semen parameters and TCS concentration using EmpowerStats software (X&Y solutions, Inc., Boston, MA, USA).

## 3. Results

Table 1 and Table 2 present the distribution of semen quality parameters and urine TCS concentration. The detectable rate of TCS was 96.7%. Semen quality parameters were comparable to those from fertile men, except that the total sperm count was slightly lower [16]. There was no significant difference between included and excluded participants in demographic characteristics such as age, body mass index (BMI), education, smoking and drinking practices (Table A1). Participants included in the final analysis had an average age of 31 years old with a median BMI of 24.0 kg·m^−2^. Among them, 27 (5.7%) men had sperm count below the reference (<39 million), 21 (4.5%) had low sperm concentration (<15 million·mL^−1^), and 126 (26.8%) had low percentage of forward moving sperms (<32% motile) according to WHO (2010, 5th Edition) reference values. Meanwhile, a total of 241 men had the four semen parameters at or above the reference. Table A2 and Table A3 show that demographic characteristics were comparable among subgroups categorized by semen quality and TCS levels.

**Figure 2:**
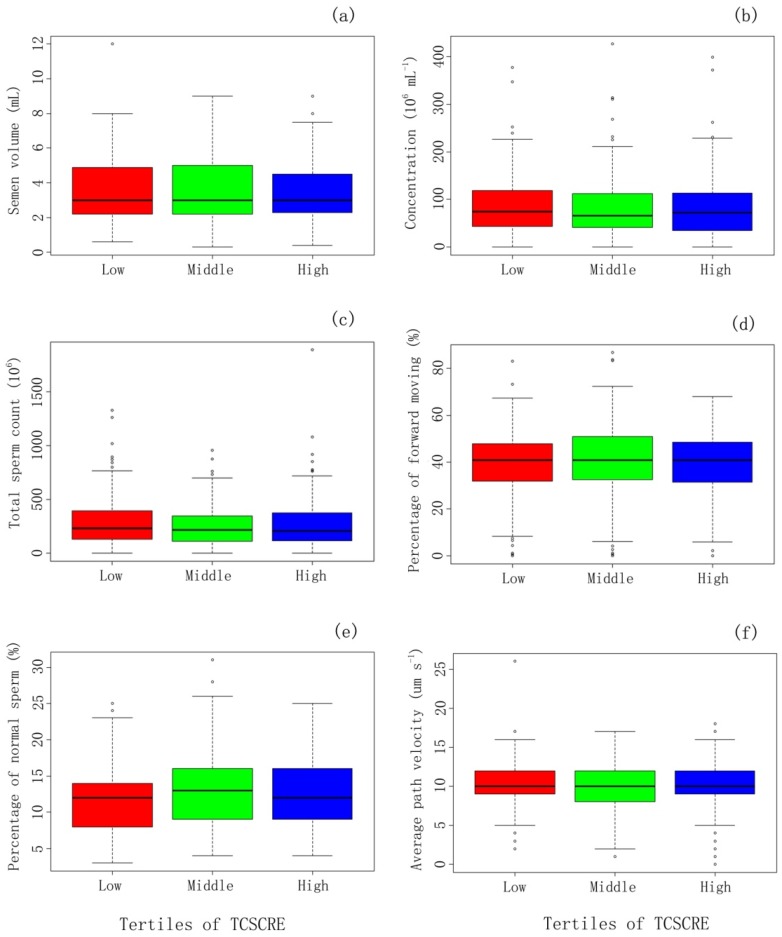
Comparison of semen quality parameters between tertiles of urinary TCS concentration among Chinese men (471) recruited during November 2013–March 2014. The *x*-axis refers to tertiles of urinary TCS level corrected by creatinine concentration (TCSCRE): low tertile (<0.66 ng·mg^−1^), middle tertile (0.66–2.33 ng·mg^−1^) and high tertile (≥2.33 ng·mg^−1^); The *y*-axis refers to sperm quality parameters, including semen volume (**a**); sperm concentration (**b**); total sperm count (**c**); percentage of forward moving (**d**); percentage of normal sperm (**e**) and average path velocity (**f**). The five lines extending vertically along Y-axis from top to bottom are respectively defined to be: Q3 (75%) + 1.5 × inter quartile range (IQR), Q3, median, Q2 (25%), Q2− 1.5 × IQR.

**Figure 3:**
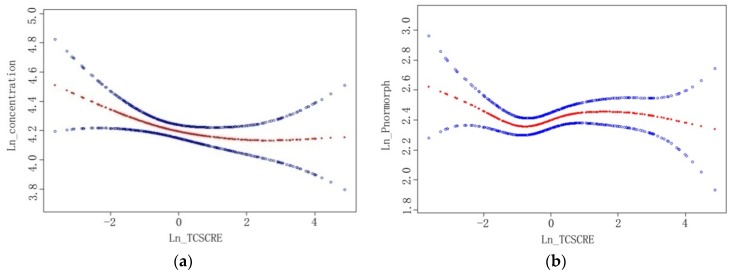
The relationships between triclosan (TCS) and sperm concentration and percent of normal morphology sperm among Chinese men (471) recruited during November 2013–March 2014. The *x*-axis refers to natural logarithm-transformed urinary TCS level corrected by creatinine concentration (Ln_TCSCRE); The*y*-axis refers to natural logarithm-transformed sperm concentration (Ln_concentration) (**a**) and percent of normal morphology sperm (Ln_Pnormorph) (**b**). The red line is fitted by generalized additive model showing the relationship between *x* and *y* axes. The two blue lines refer to 95% confidence intervals. All models were adjusted for age, BMI, abstinence period, education, income, current smoking, drinking and exposure to other chemicals or heavy metals.

Box-plot diagrams show no significant difference in semen parameters among tertiles of urine TCS concentration (Figure 2). Table 3 presents the associations of semen quality parameters and TCS concentration overall and in each tertile of urinary TCS level. There is an inverse linear association between natural logarithm (Ln) transformed TCS concentration (Ln-TCS) and Ln transformed number of forward moving sperms (adjusted coefficient β = −0.17; 95% confidence interval (CI) (−0.32, −0.02)). Moreover, among men with the lowest tertile of TCS level, reverse associations were found between Ln-TCS and sperm concentration (β = −0.21; 95% CI (−0.41, −0.01)), as well as total sperm count (β = −0.25; 95% CI (−0.48, −0.02)), number of forward moving sperms (β = −0.35; 95% CI (−0.68, −0.03)), percentage of normal morphologic sperms (β = −1.64; 95% CI (−3.05, −0.23)) and number of normal morphologic sperms (β = −0.48; 95% CI (−0.80, −0.16)). No significant association was found in the middle and high tertiles of urinary TCS level. Smoothing splines were applied to plot the relationship of TCS level and sperm concentration (a) and the relationship of TCS level and percentage of normal morphologic sperms (b). They showed similar relationships: sperm concentration or percentage of normal morphologic sperms decreased with increasing TCS concentration at lower TCS level but remained almost unchanged at a higher level (Figure 3).

## 4. Discussion

We found an inverse association between urine TCS concentration and the number of forward moving sperms. Among the participants with relatively low TCS burden, TCS was negatively associated with sperm concentration, sperm count, number of forward moving sperms, and percentage and number of normally morphologic sperms. This finding suggests that environmental exposure of TCS may have impact on semen quality.

To our best knowledge, this is the first report showing a significant association between environmental exposure of TCS and semen quality in humans. In a case-control study conducted in China, Chen *et al.* found no relationship between TCS exposure and idiopathic male infertility [22]. Den Hond *et al.* found no effect of TCS on total motility count decrease either [23]. Two improvements in the current study may have enabled us to find significant associations. First, the semen parameters were used as continuous variables in our statistical model, which had greater statistical power in comparison to binary outcomes [24]. Second, we modified the method of urinary TCS measurement and lowered the LOD to 0.1 μg·L^−1^, which raised the detectable rate from 48% in the previous study [22] to 96.7% in our investigation. Additionally, triclosan exposure levels in China seem to be quite low compared to European (GM = 2–3 µg·L^−1^ in Belgium [23]; GM = 6.1 µg·L^−1^ in Spain [25]) and American levels (GM = 13 µg·L^−1^ in U.S. [8]; GM = 12.3 ug·L^−1^ in Canada [19]). This forms the basis of our ability to observe the effect of TCS on a low level. The high tertile in the present study would correspond to low exposure groups in most biomonitoring studies. Working on higher exposure levels may have failed them to show the relationship between TCS exposure and decreased sperm quality.

In the present study, the most significant associations between semen quality parameters and TCS were observed only at the lower TCS levels. These associations diminished as the TCS level rose. This “non-monotonic” dose-response relationship was also observed between TCS and BMI in NHANES from 2003–2008 [26], where TCS was associated with increasing BMI. However, the association was stronger at low concentrations rather than moderate and the association is insignificant at high concentrations. TCS decreased the synthesis of androgens followed by reduced sperm production in treated male rats, and the responses were almost similar at moderate and high dose levels [12]. The pattern prompted us to suspect that the effect of TCS on semen quality might reach saturation at a certain point, so that semen quality decreases with rising TCS level at low TCS level but remains similar as TCS level continue to rise. More research is needed to elucidate the underlying mechanism. Nonetheless, our findings suggest that the threshold of TCS action on semen may be quite low and most men are sensitive to TCS at that level.

TCS is considered as an endocrine disruptor [27]. At a relative high dose (above 10 mg/kg/day), it decreases both serum gonadotropin hormones and testosterone [12], and mimics estrogen activities by enhancing estrogen receptor (ER) response at a moderate exposure level (less than 5 mg/kg/day) [28,29]. The strength of the latter action is dependenton estrogen concentration [28,29]. For instance, TCS at as low as 4.69 mg·kg^−1^ in combination with a single dose of ethinylestrodial (EE) resulted in a significant increase in rat uterine weight while at as high as 300 mg·kg^−1^ without cotreatment of estrogen, TCS did not alter the uterine weight [28]. ERs play a role in regulating testicular function and spermatogenesis [30,31,32]. If environmental exposure of TCS affects semen quality by enhancing the effect of estrodial through coactivating ER, considering low level of estrodial in men, small changes could cause relatively large percent of fluctuation. It is possible that low environmental exposure could cause mild effects. TCS at environmental concentrations enhances a feminising effect of oestrogen instead of active as antiandrogen in male fish [33], which supports our hypothesis. Besides, TCS has also exhibited a tendency to accumulate in the epididymis [13] where ERs are significantly expressed [34].

In addition to co-activator of ER, some other cell-based essays revealed the interaction of TCS and androgen receptor (AR), including antiandrogenicity [9,10,24,35] and androgenicity [36,37]. The findings are divergent, but this is not surprising. Steroid receptors exhibit some degree of promiscuity toward xenobiotics. High enough concentrations of xenobiotics with weak affinity could interact with the hormone receptor binding pocket and evoke a response [38,39]. However, AR receptor-mediated agonistic or antagonisticeffects of TCS have yet to be reported *in vivo*.

Several limitations of our study need to be kept in mind. First, due to the variability of TCS levels in spot urine, a single urine sample may not be representative of the usual environmental exposure level of an individual. However, it has been found that triclosan concentrations in a single urine sample could be used to categorize the six-month average exposure to triclosan in 6–10 year-old children [40]. Although we cannot readily generalize this finding in children to adults, we may speculate that adult habitual lifestyle and body composition are much more stable than children. Thus, the TCS level in the spot urine sample may represent individual’s general exposure level. Likewise, we collected only one single semen sample from each volunteer to represent his semen quality. The variability may have undermined the precision of our results. However, studies have shown that within-subject fluctuations of semen quality are smaller than between-subject variability [41]. Thus, the conclusion may still be valid if a study has a sufficient number of people [42]. Second, since this is a cross-sectional study, a causal inference may not be made between TCS and semen quality and reverse causation cannot be excluded. Third, our volunteers were from a reproductive health clinic. They may not be representative of the general population. Finally, we have no data on hormone levels, with which we could explain the effect of TCS on semen quality more comprehensively.

## 5. Conclusions

An association between environmental exposure to TCS and poor semen quality parameters was observed in this cross-sectional study, suggesting that TCS may affect human sperm production and normal morphology. However, the association was limited to the lowest tertile. Additional studies are needed to confirm or refute our findings.

## Figures and Tables

**Table 1:** Distribution of semen quality parameters in the present study.

Semen Parameter	*N*	Mean	Selected Percentiles				
			10th	25th	50th	75th	90th
Semen volume (mL)	471	3.5	1.6	2.2	3.0	4.9	6.0
Concentration (10^6^ mL^−1^)	471	85.7	24.9	40.1	70.8	115.6	163.7
Total sperm count (10^6^)	471	275.9	63.7	120.8	217.2	377.6	550.0
Sperm motility (Moving forward %)	464	39.5	19.4	31.9	40.8	49.0	55.7
Sperm morphology (Normal %)	380	12.3	6.0	8.0	12.0	16.0	18.9
VAP (Average path Velocity μm·s^−1^)	458	10.1	6.9	9.0	10.0	12.0	14.0

**Table 2:** Distribution of urinary triclosan (TCS) concentration in the present study.

EED	PercentDetected	GM	LOD	Percentile						Maximum
				10th	25th	50th	75th	90th	95th	
TCS(ng·mL^−1^)	96.4	1.12	0.1	0.20	0.50	1.12	3.38	14.35	33.37	98.01
TCS_Cre(ng·mg^−1^)	96.4	0.99		0.21	0.41	0.97	2.95	12.23	21.13	131.22

TCS_Cre: creatinine-corrected urinary TCS concentration; GM: Geometric mean; LOD: limit of detection.

**Table 3:** Associations between semen parameters and urinary TCS level corrected by creatinine concentration (TCS_Cre) in each TCS_Cre tertile level.

Semen Parameter	TCS_Cre ^a^							
	1st Tertile (<0.66 ng·mg^−1^)		2nd Tertile (0.66–2.33 ng·mg^−1^)		3rd Tertile (≥2.33 ng·mg^−1^)		Total *N* = 471	
	Adjusted-β ^b^ Coefficient (95% CI)	*p*-Value	Adjusted-β ^b^ Coefficient (95% CI)	*p*-Value	Adjusted-β ^b^ Coefficient (95% CI)	*p*-Value	Adjusted-β ^b^ Coefficient (95% CI)	*p*-Value
**Sperm volume (mL)**	−0.16 (−0.63, 0.32)	0.51	−0.28 (−1.07, 0.52)	0.50	−0.24 (−0.50, 0.03)	0.08	−0.20 (−0.43, 0.02)	0.07
**Concentration ^a^ (10^6^ mL^−1^)**	−0.21 (−0.41, −0.01)	0.04	0.15 (−0.19, 0.49)	0.40	0.04 (−0.09, 0.16)	0.58	−0.01 (−0.11, 0.08)	0.77
**Total sperm count ^a^ (10^6^)**	−0.25 (−0.48, −0.02)	0.04	0.03 (−0.32, 0.39)	0.85	−0.06 (−0.19, 0.08)	0.40	−0.09 (−0.20, 0.01)	0.09
**Sperm motility**								
Percentage of forward moving (%)	−0.99 (−4.74, 2.76)	0.60	0.66 (−6.42, 7.74)	0.86	−1.55 (−3.94, 0.84)	0.20	−1.19 (−3.11, 0.72)	0.22
Number of forward moving (10^6^) ^a^	−0.35 (−0.68,−0.03)	0.04	−0.18 (−0.72, 0.35)	0.50	−0.12 (−0.28, 0.04)	0.16	−0.17 (−0.32,−0.02)	0.02
**Sperm morphology**								
Percentage of normal (%)	−1.64 (−3.05,−0.23)	0.02	2.76 (0.25, 5.28)	0.03	−0.13 (−0.96, 0.70)	0.76	−0.39 (−1.09, 0.32)	0.28
Number of normal (10^6^) ^a^	−0.48 (−0.80,−0.16)	<0.01	0.25 (−0.24, 0.74)	0.31	−0.06 (−0.23, 0.12)	0.54	−0.14 (−0.28, 0.01)	0.06
**VAP (average path Velocity μm·s^−1^)**	−0.24 (−1.03, 0.56)	0.56	0.36 (−1.02, 1.75)	0.61	0.02 (−0.46, 0.51)	0.93	0.03 (−0.36, 0.42)	0.88

**^a^** Natural logarithm transformed; **^b^** Coefficient was adjusted for age, BMI, abstinence period, education, income, current smoking, drinking and exposure to other chemicals or heavy metals.

## Supplementary Materials

The following are available online at www.mdpi.com/1660-4601/13/2/224/, Table S1: Did you come into contact with the following material in the nearly one year?

## Appendix

**Table A1:** Distribution of demographic characteristics between included and excluded volunteers.

Demographic Characteristics	Included	Excluded	*p* Value
	*N* = 471	*N* = 55	
Age (Years) mean ± SD	30.8 ± 4.1	31.1 ± 3.5	0.69
BMI (Kg·m^−2^) mean ± SD	24.0 ± 3.1	24.0 ± 3.3	0.93
Education (Years)			0.91
≤15	129 (27.4%)	13 (23.6%)	
16	232 (49.3%)	30 (54.5%)	
≥17	110 (23.4%)	12 (21.8%)	
Income (¥10^4^/y)			0.97
<10	161 (34.2%)	19 (34.5%)	
10–30	234 (49.7%)	28 (50.9%)	
>30	42 (8.9%)	5 (9.1%)	
Refuse to answer	30 (7.2%)	3 (5.5%)	
Current Smoking (No./day)			0.11
0	361 (76.6%)	44 (80.0%)	
1–10	83 (17.7%)	5 (9.1%)	
>10	27 (5.7%)	6 (10.9%)	
Drinking ^a^			0.83
Never	164 (34.8%)	20 (36.4%)	
Seldom	281 (59.7%)	33 (60.0%)	
Frequent	26 (5.5%)	2 (3.6%)	
Abstinence time (days)			0.99
≤3	138 (29.4%)	12 (30.0%)	
>3, ≤5	154 (32.8%)	13 (32.5%)	
>5	178 (37.9%)	15 (37.5%)	

^a^ Drinking status definition: Never (<1/month), Seldom (≥1/month, <1/week), Frequent (≥1/week).

**Table A2:** Distribution of baseline characteristics by creatinine-corrected urinary triclosan (TCS) concentration (TCS_Cre).

Demographic Characteristics	TCS_Cre	
	≤0.97 (ng·mg^−1^) *N* = 236	>0.97 (ng·mg^−1^) *N* = 235
Age (Years) mean ± SD	30.8 ± 4.1	30.9 ± 4.2
BMI (Kg·m^−2^) mean ± SD	24.0 ± 3.0	24.0 ± 3.2
**Education** (Years)		
≤15	70 (29.7)	56 (23.8)
16	118 (50.0)	118 (50.2)
≥17	48 (20.3)	61 (26.0)
**Income** (¥10^4^/y)		
<10	90 (38.1)	68 (28.9)
10–30	114 (48.3)	121 (51.5)
>30	19 (8.1)	24 (10.2)
Refuse to answer	13 (5.5)	22 (9.4)
**Current Smoking** (No./day)		
0	177 (75.0)	184 (78.3)
1–10	46 (19.5)	37 (15.7)
>10	13 (5.5)	14 (6.0)
**Drinking** ^a^		
Never	76 (32.5)	88 (37.0)
Seldom	145 (61.6)	136 (58.0)
Frequent	15 (5.9)	11 (5.0)
**Abstinence Time** (days)		
≤3	67 (28.4)	72 (30.6)
>3, ≤5	83 (35.2)	71 (30.2)
>5	86 (36.4)	92 (39.2)

^a^ Drinking status definition: Never (<1/month), Seldom (≥1/month, <1/week), Frequent (≥1/week).

**Table A3:** Distribution of baseline characteristics by semen parameters.

Demographic Characteristics	Semen Parameters			
	Normal ^a^ (*N* = 275)	Sperm Count ^b^ <39 Million (*N* = 27)	Sperm Concerntration ^b^ <15 Million/mL (*N* = 21)	Percentage of Moving Forward Sperm ^b^ <32% (*N* = 117)
Age (Years) mean ± SD	30.6 ± 4.0	30.3 ± 3.9	31.4 ± 4.8	31.5 ± 4.8
BMI (Kg·m^−2^) mean ± SD	24.2 ± 3.2	23.7 ± 2.6	23.7 ± 2.6	23.3 ± 2.9
**Education** (Years)				
≤15	72 (26.2)	8(29.6)	6 (28.6)	35 (29.9)
16	143 (52.0)	8(29.6)	6 (28.6)	52 (44.4)
≥17	60 (21.8)	11(40.7)	9 (42.9)	30 (25.6)
**Income** (¥10^4^/y)				
<10	87 (31.6)	11 (40.7)	6 (28.6)	40 (34.2)
10–30	136 (49.5)	12 (44.4)	12 (57.1)	65 (55.6)
>30	29 (10.6)	2 (7.4)	1 (4.8)	4 (3.4)
Refuse to answer	23 (8.4)	2 (7.4)	2 (9.5)	8 (6.8)
**Current Smoking** (No./day)				
0	213 (77.5)	21 (77.9)	20 (95.2)	91 (77.8)
1–10	44 (16.0)	5 (6.2)	1 (4.8)	22 (18.8)
>10	18 (6.6)	1 (9.6)	0 (0.0)	4 (3.4)
**Drinking** ^c^				
Never	90 (32.7)	7 (26.0)	8 (38.1)	47 (40.2)
Seldom	171 (62.2)	20 (74.1)	13 (61.9)	62 (53.0)
Frequent	14 (5.1)	0 (0.0)	0 (0.0)	8 (6.8)
**Abstinence Time** (days)				
≤3	86 (31.3)	14 (51.9)	10 (47.6)	32 (27.4)
>3, ≤5	92 (33.5)	4 (14.8)	5 (23.8)	36 (30.8)
>5	97 (35.3)	9 (33.3)	6 (28.6)	49 (41.9)

**^a^** All of the four sperm parameters (concentration, motility, morphology and total number) above the WHO reference levels; **^b^** A subject may contribute data to more than one category; **^c^** Drinking status definition: Never (<1/month), Seldom (≥1/month, <1/week), Frequent (≥1/week).

## References

[1] Rodricks J.V., Swenberg J.A., Borzelleca J.F., Maronpot R.R., Shipp A.M. (2010). Triclosan: A critical review of the experimental data and development of margins of safety for consumer products. Crit. Rev. Toxicol..

[2] Chalew T.E., Halden R.U. (2009). Environmental exposure of aquatic and terrestrial biota to triclosan and triclocarban. J. Am. Water Works Assoc..

[3] Reiss R., Lewis G., Griffin J. (2009). An ecological risk assessment for triclosan in the terrestrial environment. Environ. Toxicol. Chem./SETAC.

[4] Moss T., Howes D., Williams F.M. (2000). Percutaneous penetration and dermal metabolism of triclosan (2,4, 4′-trichloro-2′-hydroxydiphenyl ether). Food Chem. Toxicol..

[5] Sandborgh-Englund G., Adolfsson-Erici M., Odham G., Ekstrand J. (2006). Pharmacokinetics of triclosan following oral ingestion in humans. J. Toxicol. Environ. Health Part A.

[6] Geens T., Neels H., Covaci A. (2009). Sensitive and selective method for the determination of bisphenol-a and triclosan in serum and urine as pentafluorobenzoate-derivatives using gc-ecni/ms. J. Chromatogr. B Anal. Technol. Biomed. Life Sci..

[7] Allmyr M., Adolfsson-Erici M., McLachlan M.S., Sandborgh-Englund G. (2006). Triclosan in plasma and milk from swedish nursing mothers and their exposure via personal care products. Sci. Total Environ..

[8] Calafat A.M., Ye X., Wong L.Y., Reidy J.A., Needham L.L. (2008). Urinary concentrations of triclosan in the U.S. Population: 2003–2004. Environ. Health Perspect..

[9] Gee R.H., Charles A., Taylor N., Darbre P.D. (2008). Oestrogenic and androgenic activity of triclosan in breast cancer cells. J. Appl. Toxicol..

[10] Kumar V., Balomajumder C., Roy P. (2008). Disruption of lh-induced testosterone biosynthesis in testicular leydig cells by triclosan: Probable mechanism of action. Toxicology.

[11] Forgacs A.L., Ding Q., Jaremba R.G., Huhtaniemi I.T., Rahman N.A., Zacharewski T.R. (2012). Bltk1 murine leydig cells: A novel steroidogenic model for evaluating the effects of reproductive and developmental toxicants. Toxicol. Sci.: Off. J. Soc. Toxicol..

[12] Kumar V., Chakraborty A., Kural M.R., Roy P. (2009). Alteration of testicular steroidogenesis and histopathology of reproductive system in male rats treated with triclosan. Reprod. Toxicol..

[13] Lan Z., Hyung Kim T., Shun Bi K., Hui Chen X., Sik Kim H. (2015). Triclosan exhibits a tendency to accumulate in the epididymis and shows sperm toxicity in male sprague-dawley rats. Environ. Toxicol..

[14] Zhao S.-S., Zhang J., Tian Y. (2015). Establishment and application of determination method for BPA, TCS and 4-n-NP in urine by HPLC-MS/MS. J. Environ. Occup. Med..

[15] Lu J.C., Huang Y.F., Lu N.Q. (2010). Who laboratory manual for the examination and processing of human semen: Its applicability to andrology laboratories in china. Natl. J. Androl..

[16] Mendiola J., Jorgensen N., Andersson A.M., Calafat A.M., Ye X., Redmon J.B., Drobnis E.Z., Wang C., Sparks A., Thurston S.W. (2010). Are environmental levels of bisphenol a associated with reproductive function in fertile men?. Environ. Health Perspect..

[17] Li D.K., Zhou Z., Miao M., He Y., Wang J., Ferber J., Herrinton L.J., Gao E., Yuan W. (2011). Urine bisphenol-a (BPA) level in relation to semen quality. Fertil. Steril..

[18] Kleinbaum D.G., Muller K.E., Nizam A. (1998). Selecting the best regression equation. Applied Regression Analysis and Other Multivariate Methods.

[19] Arbuckle T.E., Marro L., Davis K., Fisher M., Ayotte P., Belanger P., Dumas P., LeBlanc A., Berube R., Gaudreau E. (2015). Exposure to free and conjugated forms of bisphenol a and triclosan among pregnant women in the mirec cohort. Environ. Health Perspect..

[20] Jurewicz J., Radwan M., Sobala W., Ligocka D., Radwan P., Bochenek M., Hanke W. (2014). Lifestyle and semen quality: Role of modifiable risk factors. Syst. Biol. Reprod. Med..

[21] Li Y., Lin H., Li Y., Cao J. (2011). Association between socio-psycho-behavioral factors and male semen quality: Systematic review and meta-analyses. Fertil. Steril..

[22] Chen M., Tang R., Fu G., Xu B., Zhu P., Qiao S., Chen X., Qin Y., Lu C., Hang B. (2013). Association of exposure to phenols and idiopathic male infertility. J. Hazard. Mater..

[23] Den Hond E., Tournaye H., De Sutter P., Ombelet W., Baeyens W., Covaci A., Cox B., Nawrot T.S., Van Larebeke N., D’Hooghe T. (2015). Human exposure to endocrine disrupting chemicals and fertility: A case-control study in male subfertility patients. Environ. Int..

[24] Ahn K.C., Zhao B., Chen J., Cherednichenko G., Sanmarti E., Denison M.S., Lasley B., Pessah I.N., Kultz D., Chang D.P. (2008). *In vitro* biologic activities of the antimicrobials triclocarban, its analogs, and triclosan in bioassay screens: Receptor-based bioassay screens. Environ. Health Perspect..

[25] Casas L., Fernandez M.F., Llop S., Guxens M., Ballester F., Olea N., Irurzun M.B., Rodriguez L.S., Riano I., Tardon A. (2011). Urinary concentrations of phthalates and phenols in a population of Spanish pregnant women and children. Environ. Int..

[26] Lankester J., Patel C., Cullen M.R., Ley C., Parsonnet J. (2013). Urinary triclosan is associated with elevated body mass index in NHANES. PLoS ONE.

[27] Huang H., Du G., Zhang W., Hu J., Wu D., Song L., Xia Y., Wang X. (2014). The *in vitro* estrogenic activities of triclosan and triclocarban. J. Appl. Toxicol..

[28] Stoker T.E., Gibson E.K., Zorrilla L.M. (2010). Triclosan exposure modulates estrogen-dependent responses in the female Wistar rat. Toxicol. Sci.: Off. J. Soc. Toxicol..

[29] Louis G.W., Hallinger D.R., Stoker T.E. (2013). The effect of triclosan on the uterotrophic response to extended doses of ethinyl estradiol in the weanling rat. Reprod. Toxicol..

[30] Saunders P.T., Sharpe R.M., Williams K., Macpherson S., Urquart H., Irvine D.S., Millar M.R. (2001). Differential expression of oestrogen receptor alpha and beta proteins in the testes and male reproductive system of human and non-human primates. Mol. Hum. Reprod..

[31] Aschim E.L., Giwercman A., Stahl O., Eberhard J., Cwikiel M., Nordenskjold A., Haugen T.B., Grotmol T., Giwercman Y.L. (2005). The rsai polymorphism in the estrogen receptor-beta gene is associated with male infertility. J. Clin. Endocrinol. Metab..

[32] Schulze C. (1988). Response of the human testis to long-term estrogen treatment: Morphology of sertoli cells, leydig cells and spermatogonial stem cells. Cell Tissue Res..

[33] Lange A., Sebire M., Rostkowski P., Mizutani T., Miyagawa S., Iguchi T., Hill E.M., Tyler C.R. (2015). Environmental chemicals active as human antiandrogens do not activate a stickleback androgen receptor but enhance a feminising effect of oestrogen in roach. Aquat. Toxicol..

[34] Couse J.F., Lindzey J., Grandien K., Gustafsson J.A., Korach K.S. (1997). Tissue distribution and quantitative analysis of estrogen receptor-alpha (ERalpha) and estrogen receptor-beta (ERbeta) messenger ribonucleic acid in the wild-type and eralpha-knockout mouse. Endocrinology.

[35] Chen J., Ahn K.C., Gee N.A., Gee S.J., Hammock B.D., Lasley B.L. (2007). Antiandrogenic properties of parabens and other phenolic containing small molecules in personal care products. Toxicol. Appl. Pharmacol..

[36] Christen V., Crettaz P., Oberli-Schrammli A., Fent K. (2010). Some flame retardants and the antimicrobials triclosan and triclocarban enhance the androgenic activity *in vitro*. Chemosphere.

[37] Foran C.M., Bennett E.R., Benson W.H. (2000). Developmental evaluation of a potential non-steroidal estrogen: Triclosan. Mar. Environ. Res..

[38] Eick G.N., Colucci J.K., Harms M.J., Ortlund E.A., Thornton J.W. (2012). Evolution of minimal specificity and promiscuity in steroid hormone receptors. PLoS Genet..

[39] Witorsch R.J. (2002). Endocrine disruptors: Can biological effects and environmental risks be predicted?. Regul. Toxicol. Pharmacol..

[40] Teitelbaum S.L., Britton J.A., Calafat A.M., Ye X., Silva M.J., Reidy J.A., Galvez M.P., Brenner B.L., Wolff M.S. (2008). Temporal variability in urinary concentrations of phthalate metabolites, phytoestrogens and phenols among minority children in the united states. Environ. Res..

[41] Francavilla F., Barbonetti A., Necozione S., Santucci R., Cordeschi G., Macerola B., Francavilla S. (2007). Within-subject variation of seminal parameters in men with infertile marriages. Int. J. Androl..

[42] Stokes-Riner A., Thurston S.W., Brazil C., Guzick D., Liu F., Overstreet J.W., Wang C., Sparks A., Redmon J.B., Swan S.H. (2007). One semen sample or 2? Insights from a study of fertile men. J. Androl..

