# Supplementary material for: Environmental Exposure to Triclosan and Semen Quality
Source: Int J Environ Res Public Health. 2016 Feb 17;13(2):224. doi: 10.3390/ijerph13020224 (PMC4772244; doi:10.3390/ijerph13020224)
Supplement: Supplementary file 1 [file ijerph-13-00224-s001.pdf]

Supplementary Materials: Environmental Exposure to Triclosan and Semen Quality

Wenting Zhu, Hao Zhang, Chuanliang Tong, Chong Xie, Guohua Fan, Shasha Zhao, Xiaogang Yu, Ying Tian and Jun Zhang

Table S1. Did you come into contact with the following material in the nearly one year?

Material	Factors		
	No	Yes	Not Clear
Pesticides (against for pests, cockroach mosquito, *etc.)*	□	□	□
Bactericide (for plants)	□	□	□
Herbicide (for plants)	□	□	□
Insecticide (for plants)	□	□	□
Polishing Material	□	□	□
Paint	□	□	□
Degreasing Agent	□	□	□
Adhesives	□	□	□
Oil Cleaner	□	□	□
Soldering	□	□	□
X-ray	□	□	□
Chemical Cleaner	□	□	□
Dye	□	□	□
Pharmaceutical Industry	□	□	□
Laboratory Chemicals	□	□	□
Anesthesia or Disinfectant	□	□	□
Strong Acid or Alkali	□	□	□
lead or Other Heavy Metals	□	□	□
Other Chemicals (please write it/them down)			

© 2016 by the authors; licensee MDPI, Basel, Switzerland. This article is an open access article distributed under the terms and conditions of the Creative Commons by Attribution (CC-BY) license (http://creativecommons.org/licenses/by/4.0/).
